# Familial clustering of risk factors for cardiovascular disease among first-degree relatives of patients with chronic kidney disease in a sub-Saharan African population

**DOI:** 10.5830/CVJA-2015-041

**Published:** 2015

**Authors:** Yemi Raji, Omolara Mabayoje, Taslim Bello

**Affiliations:** Nephrology Unit, Department of Medicine, College of Medicine, University of Ibadan, Ibadan, Nigeria; Nephrology Unit, Department of Medicine, College of Medicine, University of Lagos, Idi-Araba, Lagos, Nigeria; Nephrology Unit, Department of Medicine, College of Medicine, University of Lagos, Idi-Araba, Lagos, Nigeria

**Keywords:** cardiovascular disease, chronic kidney disease, first-degree relatives, risk factors, sub-Saharan Africa

## Abstract

**Objective:**

To determine the prevalence of risk factors for cardiovascular disease (CVD) in first-degree relatives (FDRs) of patients with chronic kidney disease (CKD) in a sub-Saharan African population.

**Methods:**

This was a cross-sectional survey of 460 subjects (230 FDRs of patients with CKD and 230 healthy controls). Anthropometrics and blood pressures were measured. Spot urine and fasting venous blood samples were obtained for biochemical analysis.

**Results:**

The prevalence of hypertension, diabetes mellitus, obesity and dyslipidaemia were significantly higher in FDRs of patients with CKD compared with the controls: 56 (24.3%) vs 29 (12.6%), *p* = 0.01; 20 (8.7%) vs 6 (2.6%), *p* = 0.01; 40 (17.4%) vs 24 (10.4%), *p* = 0.03 and 171 (74.3%) vs 138 (60.0%), *p* = 0.01, respectively. Hypertension (OR, 1.65), dyslipidaemia (OR, 1.72) and albuminuria (OR, 1.61) were independently associated with being a FDR of patients with CKD.

**Conclusion:**

In this sub-Saharan African population, risk factors for CVD were more prevalent in the FDRs of patients with CKD than in healthy controls.

## Abstract

Cardiovascular disease (CVD) is receiving global attention because of its rising prevalence and its resultant high morbidity and mortality rate and the huge economic burden. It was responsible for one-third of global deaths in 2005 and it is projected that it will account for three-quarters of the deaths worldwide by 2030.^1^-^3^ In sub-Saharan Africa, a region undergoing an epidemiological transition,^2^,^4^ recent reports have suggested that CVD may be the leading cause of death.^3^,^5^

CVD is particularly prevalent among patients with chronic kidney disease (CKD). In fact, patients with CKD are more likely to die from cardiovascular diseases than from progression of renal disease.^6^ The risk of cardiovascular death in patients with end-stage renal disease (ESRD) is 10 to 100 times that in the healthy population,^6^,^7^ and many researchers consider CKD an independent risk factor for CVD in view of the changes in the cardiovascular system associated with CKD, such as endothelial dysfunction, arterial stiffening, left ventricular hypertrophy (LVH), and vascular calcification.^8^

It has been suggested that the higher prevalence of cardiovascular disease seen in patients with CKD may in part be as a result of risk factors for CVD being more prevalent in those individuals. Evidence suggests that both traditional and non-traditional cardiovascular risk factors are more common among patients with CKD than in the general population.^9^,^10^

Relatives of patients with CKD are themselves at increased risk of developing CKD.^11^ This increased risk has been hypothesised to be due to shared genetic and environmental factors.^12^ Most of these shared factors are cardiovascular risk factors, such as hypertension, diabetes, obesity and dyslipidaemia.^11^,^13^ Inserra *et al.* reported a high prevalence of common CVD risk factors among 810 first-degree relatives (FDRs) of patients with CKD; with hypertension being present in 41.8%, overweight or obesity in 62.1%, hypercholesterolaemia in 42.9%, hyperglycaemia in 5.2%, and cigarette smoking in 34.8%.13 Tsai *et al.* and Wei *et al.* both reported the prevalence of CVD risk factors to be significantly higher in FDRs of patients with CKD compared to healthy and spousal controls.^14^,^15^

FDRs of patients with CKD are not only at increased risk of developing CKD but are also at increased risk of experiencing an adverse cardiovascular event. Because many of the CVD risk factors are modifiable, identifying individuals with a higher prevalence of these risk factors would be a cost effective way of reducing the burden of cardiovascular disease, especially in resource-poor settings.^16^-^18^ FDRs of patients with CKD appear to be one such group.

There is a paucity of data, however, on the prevalence of CVD risk factors in FDRs of patients with CKD from sub-Saharan Africa. The aim of this study was to determine the prevalence of CVD risk factors in a sub-Saharan African population of FDRs of patients with CKD and compare it with a cohort of individuals with no family history of CKD.

## Methods

This was a cross-sectional study of a cohort of 460 subjects (230 FDRs of patients with CKD and 230 age- and gender-matched controls with no personal or family history of CKD) carried out between January and June 2011. The FDRs were parents, siblings or offspring of 106 consecutively presenting and consenting patients with CKD who were receiving care at the Lagos University Teaching Hospital, Lagos, south-west Nigeria. The study protocol was approved by the health research and ethics committee of the hospital and each participating individual gave written informed consent.

Recruitment into the FDR arm of the study was carried out in two phases. In the first phase of recruitment, we enrolled 106 probands who were consecutively presenting and consenting patients with CKD attending the nephrology out-patient clinic of our teaching hospital. To be eligible for recruitment in this phase, a patient had to be 18 years of age or older and give informed consent. Patients with CKD from autosomal dominant polycystic kidney disease (ADPKD) were excluded. In the second phase, we recruited FDRs of the 106 probands with CKD.

A minimum of one and a maximum of four FDRs were selected from the family of each proband. Where there were four or less eligible FDRs in the family of a proband, all of them were recruited into the study. However, where there were more than four eligible FDRs in the family of a proband, four were selected by balloting.

Individuals were eligible for recruitment into the FDR arm of the study if they were: a parent, sibling or offspring of one of the probands, were 18 years of age or older, and gave informed consent. Exclusion criteria included: age less than 18 years, presence of symptomatic urinary tract infection, on-going febrile illness, presence of heart failure, severe current illness or malignancy, and a family history of ADPKD.

For the control arm of the study, individuals who were age and gender matched with subjects in the FDR arm, and had no family or personal history of CKD were enrolled. Inclusion criteria for subjects in the control arm were: age 18 years or older, absence of personal or family history of CKD and giving informed consent. The exclusion criteria were: age less than 18 years, presence of symptomatic urinary tract infection, on-going febrile illness, heart failure, or other severe current illness or malignancy.

Information was retrieved from the study participants using an interviewer-administered structured questionnaire. Information obtained included: socio-demographic data, personal and family history of kidney disease, a history of diabetes and hypertension, current or past use of medications including herbal preparations and over-the-counter drugs. Information regarding social habits such as cigarette smoking and alcohol consumption were also retrieved.

The weight, height, waist and hip circumferences, and blood pressure were measured in each study participant. Ten millilitres each of early morning spot urine and venous blood were obtained from all participants following an overnight fast for the determination of levels of serum creatinine, fasting plasma glucose, fasting lipids and serum uric acid, and urine albumin:creatinine ratio. Glomerular filtration rate was estimated from serum creatinine using a four-variable version of the modification of diet in renal disease (MDRD) study equation.^19^

Diabetes mellitus was defined as a fasting plasma glucose level > 126 mg/dl (7 mmol/l), or diabetes mellitus diagnosed previously by a physician, or use of insulin or oral hypoglycaemic medications.^20^ Hypertension was defined as systolic BP ≥ 140 mmHg or diastolic BP ≥ 90 mmHg, hypertension previously diagnosed by a physician, or use of antihypertensive medications.^21^ Overweight was defined as body mass index (BMI) 25–29.5 kg/m^2^ and obesity was defined as BMI ≥ 30kg/m^2^.^22^ Truncal obesity was defined as waist circumference ≥ 102 cm in males and ≥ 88 cm in females.^23^ Hyperuricaemia was defined as a serum uric acid level of ≥ 7 mg/dl.^24^ Dyslipidaemia was defined as a ratio of plasma total cholesterol and high-density lipoprotein cholesterol (TC/HDL-C) > 5.^25^

Moderate alcohol drinking was defined as consumption of one drink (14 g) per day.^26^ Moderate-to-heavy cigarette smoking was defined as smoking at least six cigarettes per day.^27^

## Statistical analysis

Statistical analyses were carried out using the statistical package for social sciences (SPSS), version 17.0 (SPSS Inc, Chicago, IL). Continuous data are presented as mean ± SD and categorical variables are expressed as proportions or percentages. Independent samples t-tests were used for comparison of group means, while the chi-square test (χ2 tests) was applied for comparison of categorical variables in FDRs and controls. Multiple logistic regression analysis was used to determine CVD risk factors that were independently associated with being a FDR of a patient with CKD. Significance was set at a *p*-value less than 0.05.

## Results

The 230 FDRs comprised 25 parents (10.8%), 78 siblings (34%) and 127 offspring (55.2%). The parents were seven fathers (3.0%) and 18 mothers (7.8%), the siblings were 39 brothers (17%) and 39 sisters (17%), while the offspring were 69 sons (30.0%) and 58 daughters (25.2%). Age- and gender-matched 230 healthy adults were recruited into the control arm of the study. Table 1 shows the clinical and biochemical characteristics of the FDRs and controls. FDRs of the patients with CKD had significantly higher mean systolic blood pressure, mean diastolic blood pressure, mean body mass index, mean waist circumference and urine albumin:creatinine ratio than the controls.

**Table 1:** Comparison of measured clinical and laboratory parameters of the FDRs of patients with chronic kidney disease and the controls

*Variables*	*FDRs (n = 230)*	*Controls (n = 230)*	*p-value*
Mean age (years)	33.49 ± 12.0	33.67 ± 12.2	0.87
Mean SBP (mmHg)	116.5 ± 22.5	112.1 ± 18.1	0.02*
Mean DBP (mmHg)	74.9 ± 12.7	71.4 ± 10.5	0.01*
Mean BMI (kg/m2)	25.5 ± 5.3	23.8 ± 4.0	0.01*
Mean WC (cm)	81.8 ± 13.3	79.3 ± 11.3	0.03*
Mean HC (cm)	100.0 ± 11.3	98.4 ± 11.5	0.13
Mean SCr (μmol/l)	89.9 ± 23.4	88.3 ± 21.1	0.42
Mean FPG (mmol/l)	4.3 ± 1.1	4.3 ± 0.9	0.79
Mean SUA (μmol/l)	239.9 ± 99.4	237.4 ± 81.3	0.85
Mean TC (mg/dl)	146.5 ± 51.0	147.8 ± 40.1	0.24
(mmol/l)	(3.79 ± 1.32)	(3.83 ± 1.04)	
Mean HDL-C (mg/dl)	30.8 ± 10.5	34.7 ± 12.6	0.10
(mmol/l)	(0.8 ± 0.27)	(0.9 ± 0.33)	
Mean LDL-C (mg/dl)	106.7 ± 42.3	107 ± 38.2	0.41
(mmol/l)	(2.76 ± 1.10)	(2.77 ± 0.99)	
Mean TG (mg/dl)	95.1 ± 22.8	92.3 ± 24.3	0.06
(mmol/l)	(1.07 ± 0.26)	(1.04 ± 0.27)	
Mean eGFR (ml/min/1.73 m2)	106.6 ± 28.3	102.3 ± 25.0	0.09
Mean urine ACR	22.1 (0.5–1.406)	18.2 (0.6–1.296)	0.02*

ACR, albumin:creatinine ratio; BMI, body mass index; DBP, diastolic blood pressure; eGFR, estimated glomerular filtration rate; FDRs, first-degree relatives of patient with chronic kidney disease; FPG, fasting plasma glucose; HC, hip circumference; HDL-C, high-density lipoprotein cholesterol; LDL-C, lowdensity lipoprotein cholesterol; SCr, serum creatinine; SUA, serum uric acid; TG, triglyceride; WC, waist circumference.

Table 2 shows a comparison of the prevalence of risk factors for CVD between the FDRs of patients with CKD and the control group. The prevalence of hypertension, diabetes, obesity, dyslipidaemia, hyperuricaemia, albuminuria and reduced estimated glomerular filtration rate (eGFR) were all significantly higher among the FDRs than in the control subjects. Hypertension (OR, 1.65), dyslipidaemia (OR, 1.72) and albuminuria (OR, 1.61) are CVD risk factors that were independently associated with being a FDR of a patient with CKD (Table 3).

**Table 2:** A comparison of the frequency of risk factors for cardiovascular disease among the FDRs of patients with chronic kidney disease and the controls

*CVD risk factors*	*FDRs (n = 230) n (%)*	*Controls (n = 230) n (%)*	*Odds ratio*	*95% CI*	*p-value*
Presence of hypertension	56 (24.3)	29 (12.6)	2.23	1.33–3.76	0.01*
Presence of diabetes	20 (8.7)	6 (2.6)	3.56	1.32–10.10	0.01*
Presence of obesity	40 (17.4)	23 (10.0)	1.89	1.06–3.40	0.02*
Significant history of cigarette smoking	14 (6.1)	6 (6.2)	2.42	0.85–7.20	0.07
Presence of truncal obesity	46 (20.0)	39 (17.0)	1.22	0.74–2.02	0.40
Significant history of alcohol use	58 (25.2)	41 (17.8)	1.55	0.97–2.50	0.05
Presence of hyperuricaemia	14 (6.1)	4 (1.7)	3.66	1.10– 3.39	0.02*
Presence of dyslipidaemia	171 (74.3)	138 (60.0)	1.93	1.28–2.93	0.01*
Presence of reduced eGFR	13 (5.7)	4 (1.7)	3.38	1.01–12.50	0.03*
Presence of albuminuria	85 (37.0)	51 (22.2)	2.06	1.34–3.17	0.01*

CI, confidence interval; CVD, cardiovascular disease; FDRs, first-degree relatives of patients with chronic kidney disease; eGFR, estimated glomerular filtration rate. Moderate alcohol drinking was defined as consumption of one drink (14 g) per day. Moderate-to-heavy cigarette smoking was defined as smoking at least six cigarettes per day.

**Table 3:** Logistic regression of cardiovascular risk factors among FDRs of patients with chronic kidney disease

*CVD risk factors*	*Odds ratio*	*95% CI*	*z-statistic*	*p-value*
Presence of hypertension	1.65	1.05–2.84	1.82	0.04*
Presence of diabetes	2.37	0.89–0.50	1.72	0.08
Presence of hyperuricaemia	2.76	0.86–8.84	1.71	0.09
Presence of dyslipidaemia	1.73	1.15–2.60	2.62	0.01*
Presence of reduced eGFR	2.12	0.64–6.99	1.23	0.21
Presence of albuminuria	1.62	1.05–2.50	2.17	0.03*

FDRs, first-degree relatives; CI, confidence interval; CVD, cardiovascular disease; eGFR, estimated glomerular filtration rate.

## Discussion

Our study showed that among our sub-Saharan African cohort, as was previously reported in other populations, risk factors for cardiovascular disease were more prevalent in the FDRs of patients with CKD compared to healthy control subjects. This finding supports the phenomenon of a clustering of CVD risk factors in families of patients with CKD.

Hypertension and diabetes are two of the most important CVD risk factors worldwide. In this study, the prevalence of both conditions was significantly higher among FDRs of patients with CKD than in the control group. However, the picture was slightly different when the prevalence was compared with the national average. While the prevalence of diabetes in the FDRs in this study was significantly higher than the national average, 8.7 vs 2.3%,^28^ the prevalence of hypertension in FDRs in the study was similar to the national average values.^29^

The high prevalence of obesity among the FDRs of patients with CKD is another important CVD risk factor that deserves attention. Some communities in sub-Saharan Africa regard being overweight or obese as a sign of affluence,^30^ and so a lot of people are motivated to gain weight. The contribution of obesity to cardiovascular morbidity and mortality is significant^22^ and with the epidemiological transition taking place in sub-Saharan Africa, strategies to reduce the burden of obesity are urgently needed.

Of particular concern is the high prevalence of dyslipidaemia found in this study. Although the prevalence of dyslipidaemia was significantly higher in the FDRs of patients with CKD, the prevalence of more than 60% found in the control arm of the study suggests that this risk factor for CVD, which is not frequently assessed in resource-poor settings because of cost, may be a more serious problem than previously anticipated.

The higher prevalence of albuminuria and reduced eGFR observed in the FDRs in this study was in keeping with findings from similar studies.^11^-^15^ The higher prevalence of CVD risk factors among the FDRs of patients with CKD in this population has highlighted the need to consider this population as having an increased risk of experiencing adverse cardiovascular events, and there is a need for targeted interventions.

Our study had some limitations, including the fact that it was a cross-sectional survey, which has its own inherent weakness, such as difficulty in interpreting associations between outcome and exposure, and lack of long-term monitoring. Also, a history of hypertension and diabetes were self-reported, which may be subject to recall bias.

## Conclusion

In this sub-Saharan African population, risk factors for CVD were more prevalent in the FDRs of patients with CKD than in healthy controls.
